# MiR-146b-5p suppresses the malignancy of GSC/MSC fusion cells by targeting SMARCA5

**DOI:** 10.18632/aging.103489

**Published:** 2020-07-06

**Authors:** Haiyang Wang, Liping Tan, Xuchen Dong, Liang Liu, Qianqian Jiang, Haoran Li, Jia Shi, Xuejun Yang, Xingliang Dai, Zhiyuan Qian, Jun Dong

**Affiliations:** 1 Department of Neurosurgery, The Second Affiliated Hospital of Soochow University, Suzhou, China; 2 Department of Neurosurgery, Tianjin Medical University General Hospital, Tianjin, China; 3 Department of Neurosurgery, The First Affiliated Hospital of Anhui Medical University, Hefei, China

**Keywords:** glioma stem-like cells (GSCs), mesenchymal stem cells (MSCs), cell fusion, tumor microenvironment (TME), miR-146b-5p, SMARCA5

## Abstract

Recent studies have confirmed that both cancer-associated bone marrow mesenchymal stem cells (BM-MSCs, MSCs) and glioma stem-like cells (GSCs) contribute to malignant progression of gliomas through their mutual interactions within the tumor microenvironment. However, the exact ways and relevant mechanisms involved in the actions of GSCs and MSCs within the glioma microenvironment are not fully understood. Using a dual-color fluorescence tracing model, our studies revealed that GSCs are able to spontaneously fuse with MSCs, yielding GSC/MSC fusion cells, which exhibited markedly enhanced proliferation and invasiveness. MiR-146b-5p was downregulated in the GSC/MSC fusion cells, and its overexpression suppressed proliferation, migration and invasion by the fusion cells. SMARCA5, which is highly expressed in high-grade gliomas, was a direct downstream target of miR-146b-5p in the GSC/MSC fusion cells. miR-146b-5p inhibited SMARCA5 expression and inactivated a TGF-β pathway, thereby decreasing GSC/MSC fusion cell proliferation, migration and invasion. Collectively, these findings demonstrate that miR-146b-5p suppresses the malignant phenotype of GSC/MSC fusion cells in the glioma microenvironment by targeting a SMARCA5-regulated TGF-β pathway.

## INTRODUCTION

Glioma is the most commonly occurring primary brain tumor and is highly malignant and aggressive [1–5]. Although the comprehensive treatment regimens are being optimized continuously, the overall survival of patients with glioblastoma remains less than 15 months [6–9]. This is in part because malignant gliomas display remarkable cellular heterogenicity and harbor glioma stem-like cells (GSCs), which act as seed cells initiating tumor propagation and progression. Thus, understanding the characteristics and mechanisms of GSCs will be important for the development of more-effective antiglioma strategies. Recently, the interactions between GSCs and tumor stromal cells in the glioma microenvironment have been attracting attention as potential targets for the treatment of gliomas [10–13]. Among tumor stromal cells, tumor-associated mesenchymal stem cells (MSCs) are thought to play a key role in tumor remodeling and progression [14–17]. At present, however, the precise actions of MSCs in promoting oncogenesis and the development of gliomas are not fully understood.

Cell fusion, as occurs with fertilization, is regarded as a necessary process that contributes to the diversity of the genotypes and phenotypes of progeny cells [18]. Cell fusion is also thought to be a potential mechanism underlying tumor heterogeneity [19]. Fusion of tumor cells with their stromal cells in the tumor microenvironment (TME) leads to faster cell expansion, resistance to chemotherapy, and enhanced invasiveness and migration as compared to the parental cells [20–23]. However, there has been little study of the fusion between tumor stem cells (TSCs) and interstitial cells in the TME. The phenotypes of the resultant fusion cells and the related molecular mechanisms needs further investigation.

In the present study, therefore, we investigated the fusion of GSCs and MSCs, which contributes to glioma proliferation, invasion, and migration. Notably, our findings indicate that miR-146b-5p-mediated SMARCA5 suppression inhibits TGF-β signaling, thereby suppressing the malignant behavior of GSC/MSC fusion cells.

## RESULTS

### Primary culture of GSCs derived from clinical surgical specimens

**Figure 1:**
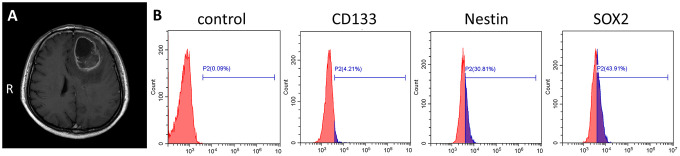
**Primary culture of human GSC-SU4s.** (**A**) Enhanced T1 MRI image of a 67-year-old male patient with left frontal mass. (**B**) Flow cytometric analysis of GSC markers on GSC-SU4 cells.

Primary human GSCs from a 67-year-old male patient diagnosed left frontal glioblastoma were cultured in medium designed to support stem cell growth (Figure 1A). We also cultured GSC-SU4 cells, which exhibited typical sphere-like cell clusters (Supplementary Figure 1A) and grew while adhering to the culture plates (Supplementary Figure 1B). Flow cytometric analysis showed the positivity rates of the GSC marker CD133, Nestin, and SOX2 among GSC-SU4 cells were 4.21%, 30.81%, and 43.91%, respectively (Figure 1B). The co-expression of GSCs markers in GSC-SU4 cells was also analyzed (Supplementary Figure 5).

### Generation of GSC-MSC fusion cells

**Figure 2:**
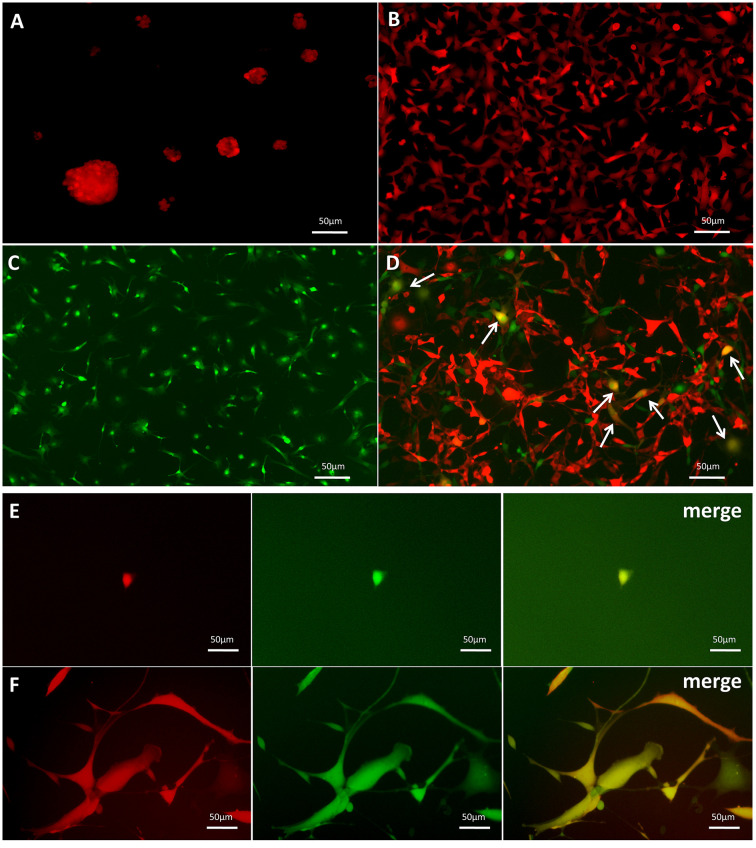
**Dual-color fluorescence tracing of co-cultured SU4-RFPs and MSC GFPs, followed by mono-cloning of double-positive fluorescent cells.** Stable expression of RFP in SU4 cells exhibiting (**A**) sphere-like or (**B**) adherent growth. (**C**) Expression of GFP in MSCs from GFP-Balb/c athymic nude mice. (**D**) RFP+/GFP+ cells (arrows) were observed in co-cultures of SU4-RFP and MSC-GFPs. (**E**) RFP+/GFP+ cells were mono-cloned from the co-cultures system and (**F**) subcultured.

GSC-SU4 cells stably expressed red fluorescent protein (SU4-RFPs) after lentivirus-mediated transfection exhibited both sphere-like clusters (Figure 2A) and adherent growth (Figure 2B). Bone marrow MSCs harvested from GFP-Balb/c mice (MSC-GFPs) were cultured in MSC medium (Figure 2C). To investigate the interaction between GSCs and MSCs, SU4-RFPs and MSC-GFPs were co-cultured at a ratio of 1:20, and RFP^+^/GFP^+^ double-positive cells (arrows) were detected after 10-14 days (Figure 2D and Supplementary Figure 2). Then these RFP^+^/GFP^+^ cells were then mono-cloned under a fluorescence microscope using the microtubule siphon method (Figure 2E) and subsequently subcultured (Figure 2F). We termed these GSC/MSC fusion cells F-GSC/MSCs.

### F-GSC/MSCs are fusion cells derived from SU4-RFPs and MSC-GFPs

**Figure 3:**
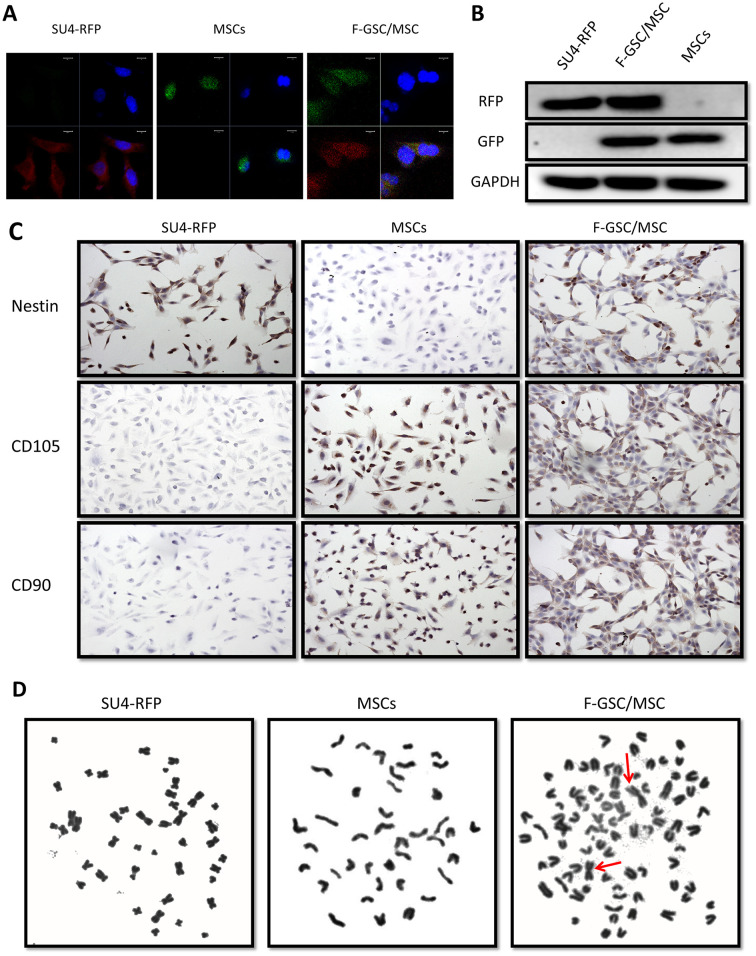
**RFP+/GFP+ cells (F-GSC/MSC) are fusion cells derived from SU4-RFPs and MSC-GFPs.** (**A**) RNA FISH and (**B**) Western blot analysis showed simultaneous expression of RFP/GFP in F-GSC/MSCs at the transcription and protein levels. (**C**) Cell surface marker identification showed that F-GSC/MSCs co-expressed markers of both GSCs and MSCs. (**D**) Human metacentric/submetacentric chromosomes (arrows) and murine telocentric chromosomes are observed in the karyotype of F-GSC/MSCs.

For further verify the fusion of MSCs and GSCs to produce F-GSC/MSCs, both transcription and translation levels of RFP/GFP genes in cells were detected using fluorescence in situ hybridization (FISH) and Western blotting. The results showed that F-GSC/MSCs co-expressed both of RFP and GFP genes, while SU4-RFPs and MSCs-GFPs expressed only RFP or GFP, respectively (Figure 3A, 3B). Immunocytochemical assays showed that F-GSC/MSCs were positive for both the GSC marker Nestin and the MSC markers CD105 and CD90 (Figure 3C). Chromosome karyotype analysis showed that the karyotype of SU4-RFPs was aneuploid with characteristics of human metacentric or submetacentric chromosomes, while the karyotype of MSC-GFPs was normal murine diploid with characteristics of murine telocentric chromosomes. The karyotype of F-GSC/MSCs harbored both human (arrows) and murine characteristic chromosome forms, and the murine telocentric chromosomes comprised the majority in the fusion cell karyotype (Figure 3D and Supplementary Figure 3). These results confirm that F-GSC/MSCs are fusion cells derived from SU4-RFPs and MSC-GFPs at the chromosome level.

### F-GSC/MSCs exhibit greater capacities for proliferation and invasion

**Figure 4:**
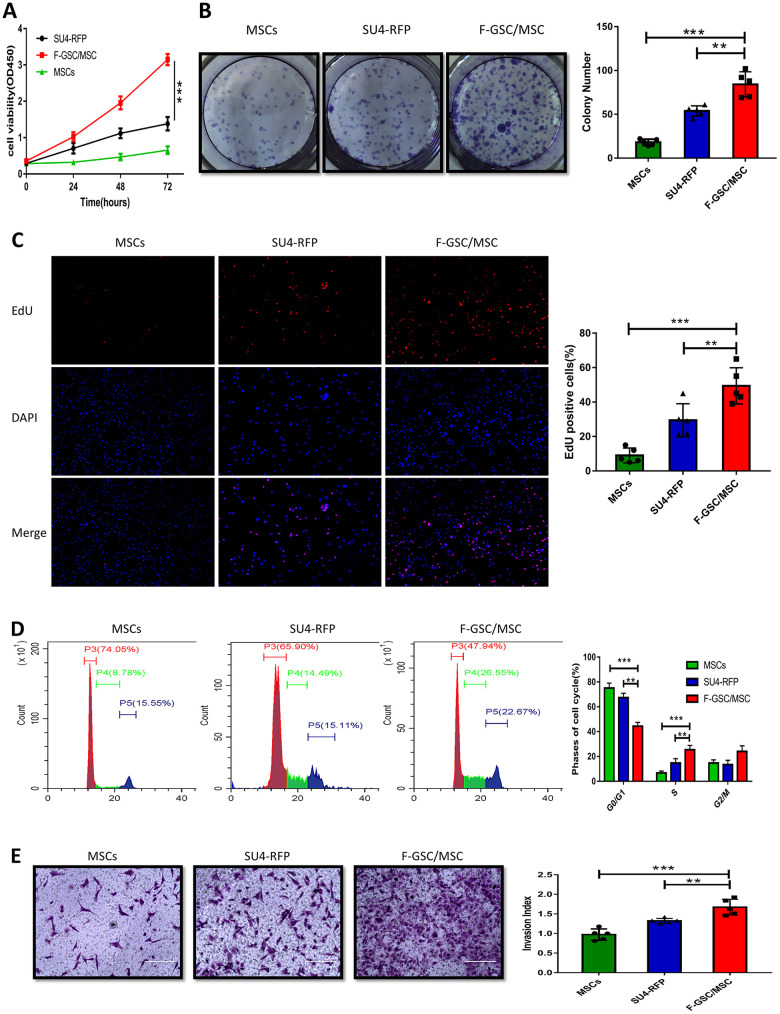
**Proliferation and invasiveness are increased in F-GSC/MSCs.** Proliferation of MSC-GFPs, SU4-RFPs and F-GSC/MSCs was measured in (**A**) CCK8 assays, (**B**) colony formation assays, (**C**) EdU assays, and (**D**) cell cycle analyses. (**E**) Invasiveness of the indicated cells was assessed with Matrigel transwell assays.

To investigate the biological characteristics of F-GSC/MSCs, the proliferation and invasiveness of F-GSC/MSCs, SU4-RFPs, and MSC-GFPs were compared. CCK8 assays revealed that F-GSC/MSCs were significantly more proliferative than their parental SU4-RFPs and MSC-GFPs (Figure 4A). Clone formation experiments and 5 ethynyl-20-deoxyuridine EDU assays showed that colony numbers and EdU-positive F-GSC/MSCs increased to a markedly greater degree than SU4-RFPs and MSCs-GFP (Figure 4B and 4C). Cell cycle analysis showed that after GSC/MSC fusion, the proportion of S phase cells increased significantly, while the proportion of G0/G1 phase cells decreased (Figure 4D). In addition, Matrigel transwell assays showed that F GSC/MSCs were significantly more invasive than SU4-RFPs or MSC-GFPs.

### Downregulation of miR-146b-5p enhances the malignancy of F-GSC/MSCs

**Figure 5:**
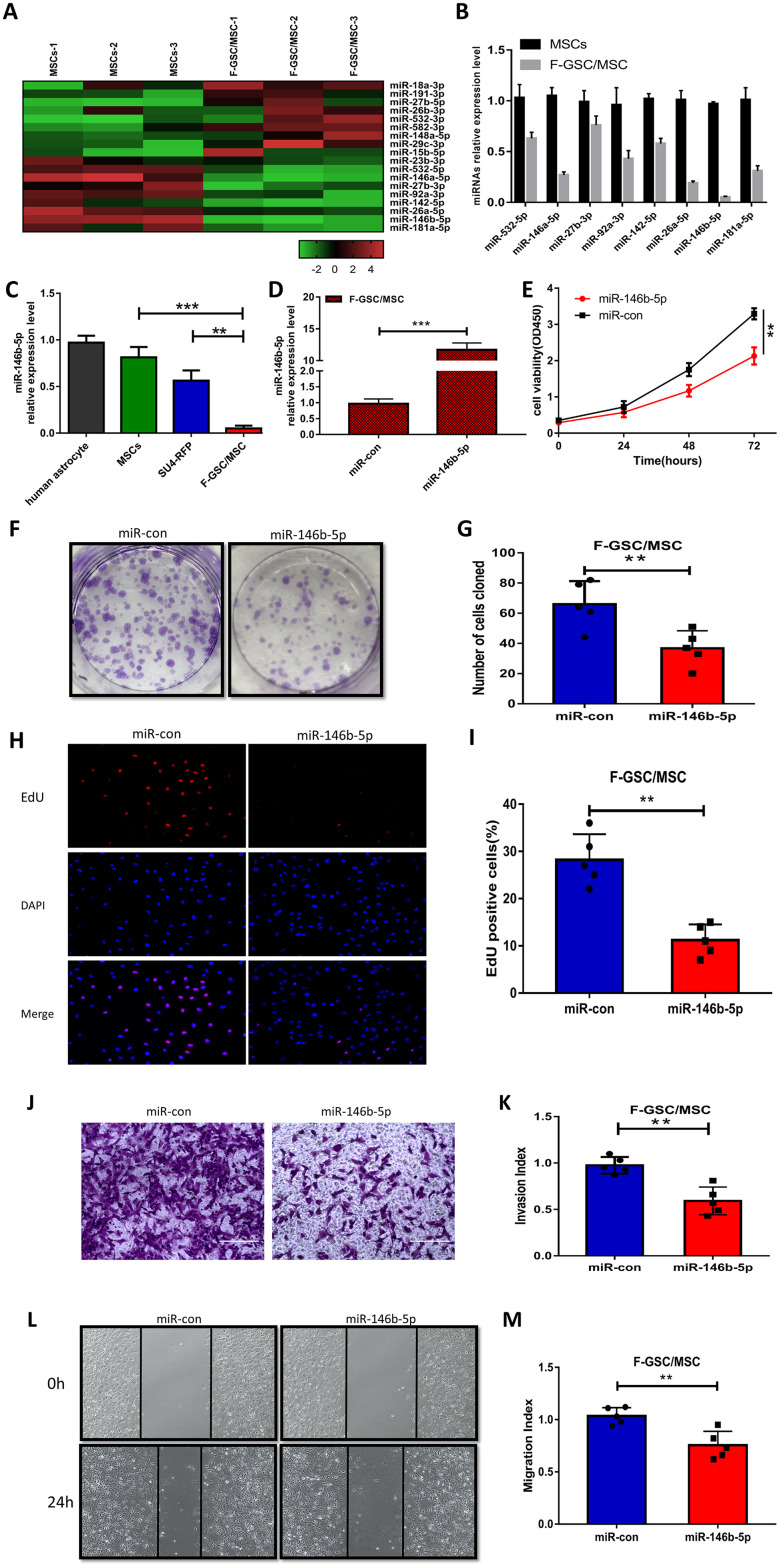
**MiR-146b-5p is downregulated in F-GSC/MSCs and its overexpression inhibits F-GSC/MSC proliferation, invasion and migration.** (**A**) Hierarchical clustering demonstrated distinguishable miRNA expression profiles in MSC and F GSC/MSC microarray data. (**B**) qPCR verified miR-146b-5p expression was the lowest among downregulated miRNAs in F-GSC/MSCs. miR-146b-5p expression in (**C**) F-GSC/MSs was lower than in MSC-GFPs or SU4-RFPs. (**D**) miR-146b-5p levels in F-GSC/MSs were upregulated by transfection of miR-146b-5p mimics. (**E**) Proliferation of F GSC/MSCs transfected with miR-146b-5p mimic or negative control was measured with CCK8 assays. (**F** and **G**) Colony formation assays with F GSC/MSCs transfected with miR-146b-5p mimic or negative control. (**H** and **I**) Effect of miR-146b-5p upregulation on F-GSC/MSC proliferation was determined using EdU assays. (**J** and **K**) Effect of miR-146b-5p upregulation on invasiveness of F GSC/MSCs was determined using Matrigel transwell assays. (**L** and **M**) Effect of miR-146b-5p on F-GSC/MSC migration was evaluated in wound-healing assays.

To evaluate the role of miRNAs in the malignant phenotype of F-GSC/MSCs, microarray analysis was used to compare the miRNA expression profiles of normal MSCs and F-GSC/MSCs (Figure 5A and Supplementary Figure 4). qPCR analysis verified that among the differentially expressed of miRNAs, eight were downregulated in F-GSC/MSCs, with miR-146b-5p exhibiting the lowest expression level (Figure 5B). In addition, qPCR also showed that miR-146b-5p expression was obviously higher in SU4-RFPs and human astrocytes than in F-GSC/MSCs (Figure 5C). This suggests the downregulation of miR-146b-5p may play a key role in enhancing the malignancy of F-GSC/MSCs. Consistent with that idea, CCK8 assays showed that upregulation of miR-146b-5p in F-GSC/MSCs achieved by transfecting miR-146b-5p mimics (Figure 5D) suppressed F-GSC/MSC proliferation (Figure 5E), colony formation (Figure 5F and 5G), and EdU incorporation (Figure 5H and 5I) as compared to controls transfected with control miRNA. In addition, Matrigel transwell assays showed that miR-146b-5p overexpression also reduced the invasiveness of F-GSC/MSCs (Figure 5J and 5K), while wound healing assays showed that miR-146b-5p overexpression inhibited recovery rates of F-GSC/MSCs (Figure 5L and 5M).

### SMARCA5 is a negatively regulated downstream target of miR-146b-5p

**Figure 6:**
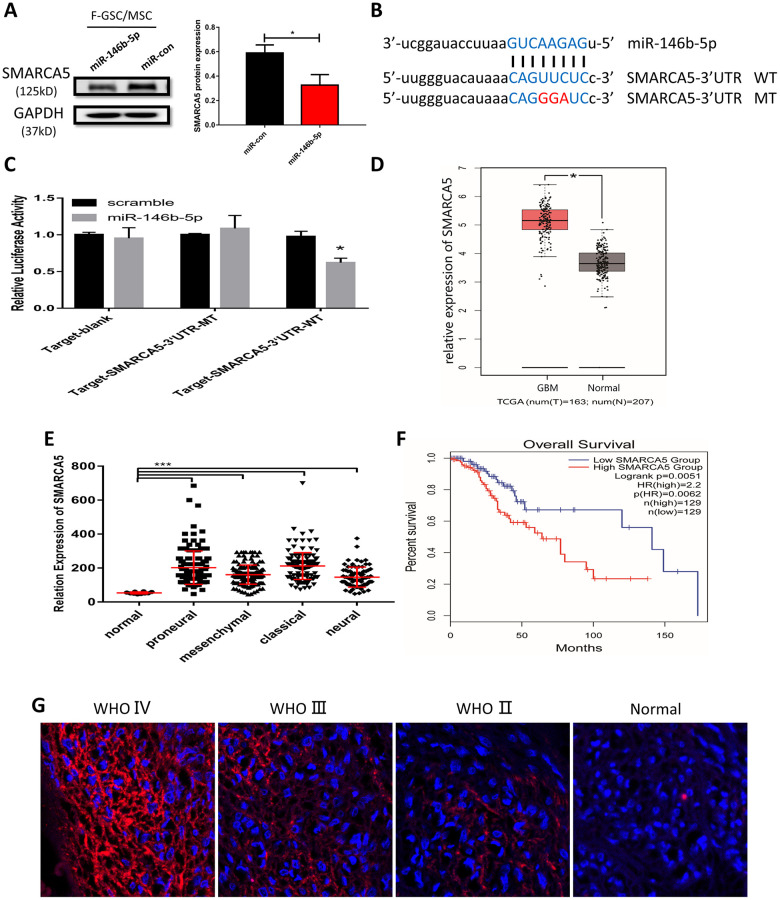
**MiR-146b-5p negatively regulates SMARCA5, which is highly expressed in high-grade gliomas.** (**A**) Western blot analysis of SMARCA5 expression in F GSC/MSCs overexpressing miR-146b-5p. (**B**) Predicted binding site between miR 146b-5p and SMARCA5. Wild type (WT) and mutant (MT) SMARCA5 vectors were constructed for luciferase assays. (**C**) Luciferase activity indicated miR-146b-5p bound directly to the 3’ UTR of SMARCA5. (**D**) SMARCA5 expression in glioblastoma and normal tissue from a TCGA dataset. (**E**) SMARCA5 expression in different glioblastoma subtypes in the TCGA dataset. (**F**) Overall survival among glioma patients in a low SMARCA5 and high SMARCA5 group. (**G**) Immunofluorescence analysis of SMARCA5 expression in different WHO grade gliomas.

To further clarify the potential mechanisms underlying the malignancy of F-GSC/MSCs, the target predictor (StarBase v3.0: http://starbase.sysu.edu.cn/) was applied to predict the possible targets of miR-146b-5p. Among the predicted candidate genes, SMARCA5 expression was dramatically reduced in F-GSC/MSCs overexpressing miR-146b-5p (Figure 6A). Bioinformatics analysis showed that SMARCA5 is a downstream mediator of miR-146b-5p whose mRNA contains a potential binding site (Figure 6B). Luciferase assays were then performed to determine whether miR-146b-5p directly binds to the 3’ untranslated region (UTR) of wild-type (WT) SMARCA5 mRNA and inhibited its expression, but had little effect on a mutant (MT) SMARCA5 vector (Figure 6C).

SMARCA5 expression was also analyzed in a dataset from The Cancer Gene Atlas (TCGA). The results showed that SMARCA5 is dramatically up-regulated in glioblastoma as compared to normal brain tissue (Figure 6D). Moreover, the TCGA dataset indicated that SMARCA5 expression in all four of the glioblastoma subtypes (classical, mesenchymal, neural, and proneural) was significantly higher than in normal controls (Figure 6E). Survival curves for gliomas showed that the survival rates among patients exhibiting high SMARCA5 expression were much poorer than among those expressing low SMARCA5 levels (Figure 6F). In addition, analysis of SMARCA5 expression in gliomas with different WHO grades showed that, consistent with the TCGA data, SMARCA5 expression in high-grade gliomas was marked higher than in low-grade gliomas and normal tissue (Figure 6G). These data suggest SMARCA5 may be novel prognostic biomarker in glioma that is directly negatively regulated by miR-146b-5p.

### SMARCA5 restoration rescued miR-146b-5p mediated inhibition of cell proliferation, invasion and migration in GSC/MSC fusion cells via the TGF-β signaling pathway

**Figure 7:**
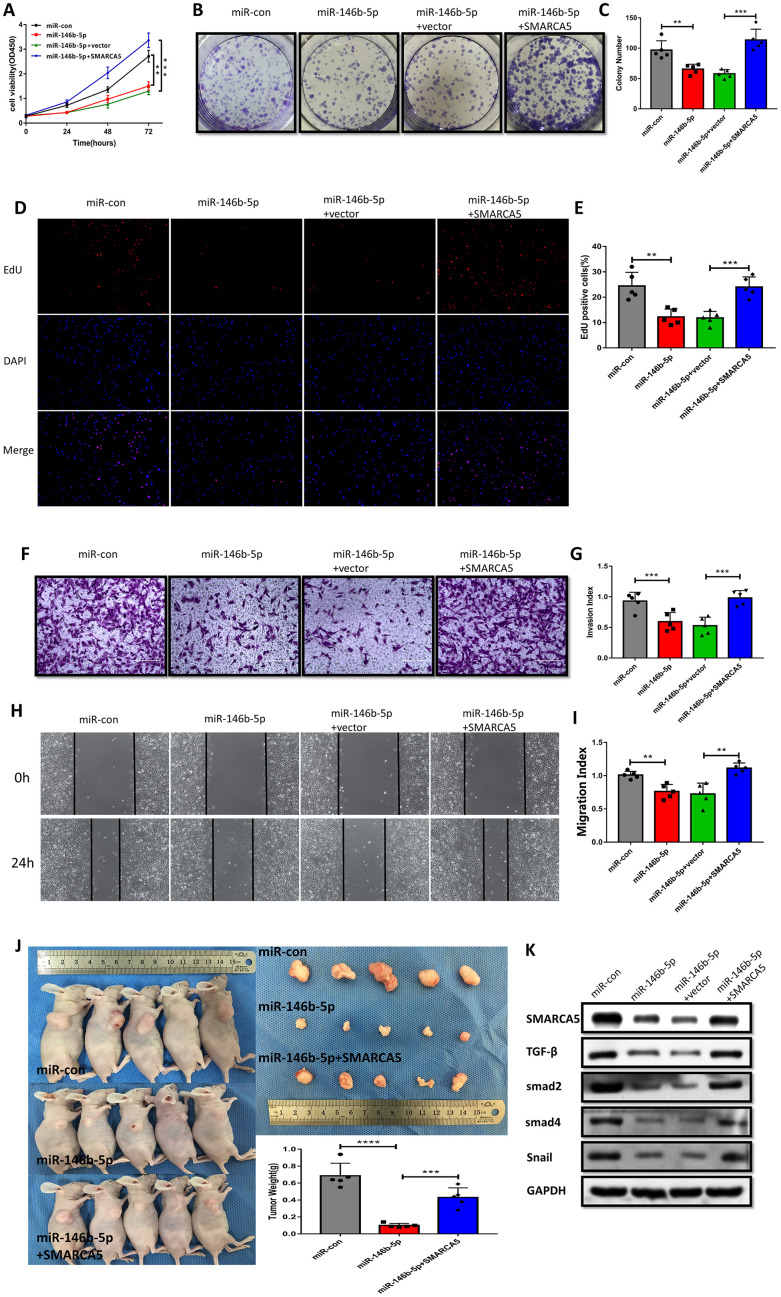
**SMARCA5 restoration reverses miR-146b-5p-mediated inhibition of F GSC/MSC proliferation and metastasis.** (**A**) CCK8, (**B** and **C**) colony formation, and (**D** and **E**) EdU assays were conducted to evaluate F GSC/MSC proliferation after transfection with miR-146b-5p alone or co-transfection with miR-146b-5p plus SMARCA5. (**F** and **G**) Transwell assays to assess the invasiveness of F GSC/MSCs transfected with miR-146b-5p alone or with miR-146b-5p plus SMARCA5. (**H** and **I**) Wound healing assays to assess migration of F GSC/MSCs transfected with miR 146b 5p alone or with miR-146b-5p plus SMARCA5. (**J**) Xenograft model for evaluation of tumorigenesis in vivo. (**K**) Western blot analysis of proteins in the TGF β pathway in F GSC/MSCs transfected with miR-146b-5p alone or with both miR 146b 5p and SMARCA5.

To further verify the impact of miR-146b-5p downregulation and the corresponding upregulation of SMARCA5 on the malignant phenotype of F GSC/MSCs, miR-146b-5p and/or SMARCA5 was overexpressed in F-GSC/MSCs, after which cell growth, migration, and invasion were evaluated both in vitro and in vivo. The results of CCK8, colony formation, and EdU assays showed that combined overexpression of SMARCA5 and miR-146b-5p could reverse the inhibitory effect of miR-146b-5p on F GSC/MSC proliferation (Figure 7A–7E). In addition, SMARCA5 expression reversed miR-146b-5p-mediated inhibition of tumor invasion and migration (Figure 7F–7I).

A F-GSC/MSC xenograft model was applied to investigate the actions of miR 146b 5p/SMARCA5 in vivo. Consistent with the in vitro observations, upregulation of both SMARCA5 and miR-146b-5p led to greater F-GSC/MSC tumor growth that was seen with upregulation of miR-146b-5p alone (Figure 7J). TGF-β signaling is known to play important roles promoting metastasis in many cancers [24, 25]. Within the tumors, F-GSC/MSCs overexpressing miR-146b-5p exhibited lower levels of TGF-β, Smad2, Smad4 protein expression (Figure 7K), as well as lower expression levels of Snail, which is involved in epithelial-mesenchymal transition (EMT) [26, 27]. Taken together, these results suggest that SMARCA5 upregulation abolishes the regulatory effects of miR-146b-5p on mediators in the TGF β pathway.

## DISCUSSION

Nearly 90% of cancer mortality is attributable to tumor invasion and metastasis [28]. Moreover, an increasing number of studies have shown that cell fusion may significantly promote cancer cells capacities for invasion and migration [29]. After spontaneous fusion between breast cancer cells and MSCs, the telomerase activity, proliferation and tumorigenicity of the fusion cells were significantly higher than those of their parental cells. Quick metastasis could occur through increased expression of metastasis-related genes, including S100A4 [30]. Hypoxia-induced apoptosis can stimulate fusion of MSCs with breast cancer cells, and the resultant fusion cells exhibit greater metastatic ability [23]. Epithelial-to-mesenchymal transition (EMT) is indispensable for tumor metastasis. Breast cancer cells trigger EMT and produce TSCs through fusion with MSCs, which increases their heterogeneity and metastatic capacity [31]. Through fusion between macrophages and breast cancer cells, expression of E-cadherin is downregulated and expression of N-cadherin, vimentin and snail are upregulated, together with increased expression of matrix metalloproteinase (MMP)-2, MMP-9, urokinase-type plasminogen activator (uPA), and S100A4. As a result, EMT emerges, leading to greater capacities for invasion and migration [32]. Similarly, fusion between lung cancer cells and MSCs leads to enhanced metastasis through EMT, with downregulation of E-cadherin and upregulation of N-cadherin, vimentin, α-SMA, and fibronectin-1. Also increased in the fusion cells was expression of EMT-related transcription factors, including Snail1, Slug, Twist1, ZEB1 and ZEB2. Gliomas undergo a process called proneural mesogenic transition (PMT), which was similar to EMT, and the resultant mesenchymal GSCs exhibit a more malignant phenotype than proneural GSCs, as they are more proliferative and invasive [33]. Based on those findings and the results of our experiments, we suggest that GSCs obtain a more malignant phenotype through fusion with MSCs via PMT, though this hypothesis remains to be tested.

Fusion cells are tumorigenic, harboring the characteristics and surface markers of both tumor cells and stromal cells [29]. When breast cancer cells fuse with breast stem cells (BSCs), the proliferation rate of the fusion cells is reportedly 1.5 times higher than that of the parental cancer cells and 10 times higher than that of the BSCs [34]. Fusion of hepatoma (HepG2) cells and embryonic stem cells (ESCs) produces more tumorigenic fusion cells, which are similar to TSCs, with enhanced expression of the TSC markers CD133, ALDH1 and CD44 [35]. MSCs can fuse with various tumor cells, and when spontaneous cell fusion occurs between lung cancer cells and bone marrow MSCs, the tumorigenicity of the resultant fusion cells is significantly enhanced, with expression of interstitial cell markers, vimentin and fibronectin [36]. After fusion of gastric cancer cells with MSCs, the fusion cells are more proliferative than their parental cells, and exhibit both stem-like properties and EMT with increased expression of both mesenchymal markers (vimentin and N cadherin) and TSC markers (CD44 and CD133) and decreased expression of E cadherin [22]. Upon fusion of MSCs with multiple myeloma cells, expression of Nanog, Sox2 and Oct4 increased significantly, and the drug resistance of the fusion cells was increased [20]. Finally, after fusion of GSCs with MSCs, the fusion cells exhibited enhanced angiogenic effects [37].

Most studies of glioma ascribe the poor prognosis and high rate of recurrence to the presence of GSCs [38–40]. They also report that the unlimited self-renewal capacity and persistent proliferation of GSCs [41–43] leads to resistance to radiotherapy and chemotherapy [44–46]. However, increasing attention is being paid to the TME [47, 48]. In the present study, we show that MSCs in the TME of gliomas could be transformed into malignant cells through fusion with GSCs, and the proliferation and metastasis of fusion cells were even greater than those of the GSCs, which may explain in part how terminally differentiated interstitial cells in the TME become cancer cells [49, 50].

A large number of studies of miRNAs and their corresponding pathways provide an avenue for exploring new markers for tumor grading, therapeutic effect evaluation, and prognosis [51, 52]. It was previously reported that miR-146b-5p can act as a tumor promoter or inhibitor in different tumors [53–56], but studies relevant to the role of miR-146b-5p in GSCs or MSCs in the glioma microenvironment were not available. In the present study, miR-146b-5p suppressed the malignancy of F-GSC/MSCs, and SMARCA5 was shown to be a downstream target gene of miR-146b-5p. SMARCA5 locates in the q31.1→q31.2 bands of chromosome 4 [57] and is regarded as a critical contributor to malignant tumors, such as gastric cancer [58], acute leukemia [59] and prostate cancer [60, 61], among others, where SMARCA5 is significantly upregulated and shown to promote cancer progression [62–64]. But its roles in GSCs the glioma microenvironment have not yet been described. In our experiments, we confirmed the role of a miR-146b-5p/SMARCA5/TGF-β axis in malignant F-GSC/MSCs in the glioma microenvironment, which may be a potential therapeutic target for treatment of glioma in the future.

A limitation of the present study is that it is based largely on GSC-MSC interactions in vitro, which may not fully reflect the situations in the real world of clinical patients. Further investigation will be needed to confirm the existence of F GSC/MSCs and the molecular mechanisms governing their behavior.

## MATERIALS AND METHODS

### Tumor specimens, experimental animals, and lentivirus fluorescence transfection

Clinical tumor tissue specimens were obtained from glioma patients treated at the Department of Neurosurgery of the Second Affiliated Hospital of Soochow University after obtaining informed consent. Primary cultured human GSCs-SU4 cells were derived from an adult male patient diagnosed with primary glioblastoma multiforme (pGBM). Balb/c nude mice expressing green fluorescent protein (GFP) were bred under specific pathogen-free conditions at our experimental animal center, as previously described [65]. All of the clinical and animal studies adhered to the rules of the Ethics Committee of the Second Affiliated Hospital of Soochow University.

Stable transfection of red fluorescent protein (RFP) gene into SU4 cells was accomplished using a RFP lentivirus (Genepharma, China). The bone marrow cavities of the femurs and tibias of GFP Balb/c nude mice were flushed using MesenPRO Medium (Gibco, US) after sacrificing the mice under general anesthesia. The mixture of bone marrow cells was purified by flow cytometry to obtain the MSCs using antibodies against CD105 and CD90 (CST, US).

### Fusion of glioma stem cells SU4-RFP and MSCs

Single-cell suspensions of SU4-RFP and MSCs were mixed at a 1:20 ratio and cultured in laminin coated plates in Nutrient Mixture F12/MesenPRO Medium (1:1, Gibco, US) including 1× B27 Supplement (Gibco, US), 20 ng/ml EGF (Gibco, US), 20 ng/ml bFGF (Gibco, US). After culture for 10-14 days, RFP/GFP double-positive (RFP^+^/GFP^+^) cells could be observed under a fluorescence microscope in the co-culture system. The RFP^+^/GFP^+^ cells were then mono-cloned from the co-culture system using micro-siphon techniques while being viewed under a fluorescence microscope. RFP^+^/GFP^+^ cells were then further subcultured and named after fusion GSC and BM MSC (F-GSC/MSC).

### Cell culture and transfection

Human astrocytes (ATCC, US) and F-GSC/MSCs were cultured in Dulbecco’s modified Eagle medium (DMEM; Hyclone, US) containing 10% fetal bovine serum (FBS) (BI, Israel). GSCs-SU4 cells were cultured in DMEM/F12 neural stem cell culture medium (Hyclone, US) supplemented with 1x B27 Supplement (Gibco, US), 20 ng/ml EGF (Gibco, US) and 20 ng/ml bFGF (Gibco, US). All cells were cultured in an incubator (SANYO, JP) at 37°C under 5% CO_2_.

For the overexpression of miR-146b-5p and SMARCA5, F-GSC/MSCs were transfected by miR-146b-5p mimics/lentivirus, SMARCA5 overexpression vector, or their respective controls (GenePharma, Shanghai, China) according to the manufacturer’s protocol.

### Flow cytometry for identification

After digestion and centrifugation, cells were re-suspended in 100 μl of phosphate buffer saline (PBS) and incubated with 3 μl primary anti-CD133, anti-Nestin, and anti-SOX2 antibodies (dilution 1:200) for 1-2 h at room temperature. The cells were then centrifuged, washed 3 times with PBS, and incubated with secondary antibody (Beyotime, China) for 1 h at room temperature. After centrifugation (1000 rpm) and 3 washes with PBS, the cells were suspended in 200 μl of PBS and analyzed using flow cytometry (BD Biosciences) with Cytexpert 2.0 software.

### Fluorescence in situ hybridization (FISH)

Cells were seeded onto coverslips in 24-well plates, cultured to 70% confluence, fixed in 4% paraformaldehyde for 20 min, then washed for 5 min. The fixed cells were then permeabilized by digestion with proteinase K for 10 min, rinsed with 2× sodium saline citrate buffer (SSC) for 5min, fixed again in 1% paraformaldehyde for 10 min, and washed twice for 5 min each with PBS. The specimens were then dehydrated through a sequential 70%, 85%, 100% ethanol series and air-dried, after which probe hybridization solution was added and the samples were incubated at 37°C overnight. The samples were then washed three times for 5 min each with 50% formamide in 2×SSC at 53°C, then washed for three times for 5 min each with 2× SSC at 65°C. Lastly, the samples were stained with DAPI solution and washed for 5 min with PBS.

### Western blotting

Total cell protein was extracted using RIPA buffer (Beyotime Biotechnology, China), after which 20-μg aliquots of protein were separated by 10% SDS-PAGE, transferred to PVDF membranes, and incubated overnight with primary antibodies against RFP (CST, US), GFP (CST, US), SMARCA5 (CST, US), TGF-β (CST, US), Smad2 (CST, US), Smad4 (CST, US), Snail (CST, US) and GAPDH (CST, US). The membranes were then incubated with secondary antibody for 1 h. Enhanced chemiluminescence was used for visualization and quantitative analysis.

### Immunocytochemical staining

Cells cultured on chamber slides were fixed for 20 min in methanol, permeabilized with 0.25% Triton X-100 (Beyotime, China), and incubated for 1 h in blocking solution. Primary antibodies against Nestin (CST, US), CD105 (CST, US), CD90 (CST, US) and SMARCA5 (CST, US) were applied for 1 h, after which secondary antibody (Beyotime, China) antibodies were applied for 30 min. Finally, the slides were developed with diaminobenzidine (DAB) and counterstained with hematoxylin.

### Chromosome karyotype analysis

After growing cells to 80% confluency, 100 μl colchicine (10 μg/ml) in 5 ml of medium were added to the culture dishes and incubated for 4-6 h. The cells were then digested and centrifuged, after which 75 mmol/L KCl was added, and the cells were incubated for 20 min before addition of 1 ml of fixation fluid (methanol:glacial acetic acid, 3:1) was added. After incubation for an additional 30 min, the cells were centrifuged, the supernatant was discarded, and 1 ml of the fixation solution was added again. The suspension was then transferred to slides, air dried, and stained with Giemsa. Chromosome karyotype was observed under microscope.

### CCK8 assays

Cells were seeded into 96-well plates at a density of 3,000 cells/well in 100 μl of DMEM. Every 24 h, 10 μl of CCK8 reagent (Dojindo, Japan) were added into each well and incubated for another 2 h at 37°C. A spectrophotometer (Tecan, Switzerland) was then used to measure the absorbance at 450 nm.

### Colony formation assays

Cells were seeded onto 6-well plates at a density of 500 cells per well. Fresh medium was replaced every 3 days. On the 10^th^ day, the medium was removed, and each well was washed twice with PBS. Thereafter, the cells were stained using 1 ml of 0.1% crystal violet, which was added to each well for 20 min. The cells were then washed three times for 3 min each with PBS, after which the plates were dried at room temperature and the colonies were counted.

### 5-Ethynyl-20-deoxyuridine (EdU) assay

Cells were seeded into 24-well plates at a density of 5×10^4^ cells/well and incubated overnight, after which 300 μl of EdU (50 μM) (RiboBio, China) were added to each well, and the cells were incubated for additional 2 h. The cells were then fixed in 4% polyformaldehyde for 20 min, permeabilized with 0.5% TritonX-100 (Beyotime, China) for 20 min, and stained in 300 μl of Apollo dye solution (RiboBio, China) for 25 min. Cell nuclei were stained with Hoechst (RiboBio, China) for 10-30 min. The proportion of EdU-positive cells were determined using a fluorescence microscope.

### Cell cycle analysis

Cells were digested, washed in PBS, and fixed in 70% cold ethanol overnight. The fixed cells were resuspended with 50 μg/ml propidium iodide (PI) (Multiscience, China) for 30 min at room temperature, after which cell cycle was analyzed using a flow cytometer (Beckman, US) with Cytexpert 2.0 software.

### Invasion assays

Transwell chambers (Corning, US) were coated with Matrigel, after which SU4 RFPs or F-GSC/MSCs in 120 μl of serum-free medium were seeded into the upper chambers at 5x10^4^ cells/chamber, and 600 μl of complete medium containing 10% FBS were added to the lower chamber. After incubation for 24 h at 37°C, the unmigrated cells on the upper surface were wiped away with cotton swabs. Cells on the lower surface were fixed for 30 min with methanol and stained with 0.1% crystal violet.

### Wound healing assay

Cells were seeded onto 6-well plates and cultured overnight at 37°C, after which wounds were made in cell monolayers using a 200-μl pipette tip. The cells were then washed with PBS and incubated in serum-free DMEM. Images of the wound area were analyzed using Image J software after 24 h (NIH, Bethesda, USA).

### Quantitative real-time reverse transcription PCR (qRT-PCR)

Total RNA was extracted from cells with TRIzol (Invitrogen, US) and reverse transcribed to cDNA using a reverse transcription kit (GenePharma, China). Levels of miRNA expression were determined using the 2^−ΔΔCt^ method. Expression of U6 served as a control.

### Luciferase reporter assay

F-GSC/MSCs were cultured in 24-well plates and co-transfected with miR 146b 5p or scramble control plus the SMARCA5 3’-UTR, its mutated 3’-UTR, or empty vector. After 48 h, luciferase assays were then performed using a Dual-Luciferase Reporter Assay System (Promega, USA).

### Immunofluorescence

Glioma tissue sections (5-μm) were fixed with 4% formaldehyde for 15 min at room temperature and blocked with blocking buffer for 60 min. The samples were then incubated first with primary anti-SMARCA5 antibody (dilution 1:100, CST, US) overnight at 4°C, then with fluorochrome-conjugated secondary antibody for 1–2h at room temperature in the dark and stained with DAPI.

### Tumorigenicity assay

Four-week-old athymic Balb/c nude mice (15-20 g) were bred in the animal center at Soochow University under specific pathogen-free conditions. F-GSC/MSCs (1×10^6^) overexpressing miR-146b-5p, miR-146b-5p + SMARCA5, or a negative control were subcutaneously injected into the right flank of each mouse. After 5 weeks, all mice were sacrificed under general anesthesia, and the tumors were excised and weighed.

### Statistical analysis

All data were expressed as the mean±SD. Groups were compared using t-tests, q tests, or one-way-ANOVA, as appropriate. All statistical analyses were performed using Prism 7.0 (GraphPad Software, US). Values of P<0.05 were considered statistically significant.

## Supplementary Material

Supplementary Figures

## References

[1] Caccese M, Indraccolo S, Zagonel V, Lombardi G. PD-1/PD-L1 immune-checkpoint inhibitors in glioblastoma: a concise review. Crit Rev Oncol Hematol. 2019; 135:128–34. 10.1016/j.critrevonc.2018.12.002 30819441

[2] Qi Q, Kang SS, Zhang S, Pham C, Fu H, Brat DJ, Ye K. Co-amplification of phosphoinositide 3-kinase enhancer a and cyclin-dependent kinase 4 triggers glioblastoma progression. Oncogene. 2017; 36:4562–72. 10.1038/onc.2017.67 28368413 PMC5552418

[3] Sachdeva R, Wu M, Smiljanic S, Kaskun O, Ghannad-Zadeh K, Celebre A, Isaev K, Morrissy AS, Guan J, Tong J, Chan J, Wilson TM, Al-Omaishi S, et al. ID1 is critical for tumorigenesis and regulates chemoresistance in glioblastoma. Cancer Res. 2019; 79:4057–71. 10.1158/0008-5472.CAN-18-1357 31292163

[4] Johung T, Monje M. Neuronal activity in the glioma microenvironment. Curr Opin Neurobiol. 2017; 47:156–61. 10.1016/j.conb.2017.10.009 29096244 PMC5927594

[5] Jung Y, Ahn SH, Park H, Park SH, Choi K, Choi C, Kang JL, Choi YH. MCP-1 and MIP-3α secreted from necrotic cell-treated glioblastoma cells promote migration/infiltration of microglia. Cell Physiol Biochem. 2018; 48:1332–46. 10.1159/000492092 30048972

[6] Chen P, Zhao D, Li J, Liang X, Li J, Chang A, Henry VK, Lan Z, Spring DJ, Rao G, Wang YA, DePinho RA. Symbiotic macrophage-glioma cell interactions reveal synthetic lethality in PTEN-null glioma. Cancer Cell. 2019; 35:868–84.e6. 10.1016/j.ccell.2019.05.003 31185211 PMC6561349

[7] Seker F, Cingoz A, Sur-Erdem İ, Erguder N, Erkent A, Uyulur F, Esai Selvan M, Gümüş ZH, Gönen M, Bayraktar H, Wakimoto H, Bagci-Onder T. Identification of SERPINE1 as a regulator of glioblastoma cell dispersal with transcriptome profiling. Cancers (Basel). 2019; 11:1651. 10.3390/cancers11111651 31731490 PMC6896086

[8] Li M, Long S, Hu J, Wang Z, Geng C, Ou S. Systematic identification of lncRNA-based prognostic biomarkers for glioblastoma. Aging (Albany NY). 2019; 11:9405–23. 10.18632/aging.102393 31692451 PMC6874448

[9] Takashima Y, Kawaguchi A, Yamanaka R. Promising prognosis marker candidates on the status of epithelial-mesenchymal transition and glioma stem cells in glioblastoma. Cells. 2019; 8:1312. 10.3390/cells8111312 31653034 PMC6912254

[10] Chen X, Hu L, Yang H, Ma H, Ye K, Zhao C, Zhao Z, Dai H, Wang H, Fang Z. DHHC protein family targets different subsets of glioma stem cells in specific niches. J Exp Clin Cancer Res. 2019; 38:25. 10.1186/s13046-019-1033-2 30658672 PMC6339410

[11] Hide T, Komohara Y, Miyasato Y, Nakamura H, Makino K, Takeya M, Kuratsu JI, Mukasa A, Yano S. Oligodendrocyte progenitor cells and macrophages/microglia produce glioma stem cell niches at the tumor border. EBioMedicine. 2018; 30:94–104. 10.1016/j.ebiom.2018.02.024 29559295 PMC5952226

[12] Yan GN, Yang L, Lv YF, Shi Y, Shen LL, Yao XH, Guo QN, Zhang P, Cui YH, Zhang X, Bian XW, Guo DY. Endothelial cells promote stem-like phenotype of glioma cells through activating the hedgehog pathway. J Pathol. 2014; 234:11–22. 10.1002/path.4349 24604164 PMC4260128

[13] Ji XY, Ma JW, Dong J. Myeloid-derived suppressor cells and nonresolving inflammatory cells in glioma microenvironment: molecular mechanisms and therapeutic strategies. Glioma. 2018; 1:2–8. 10.4103/glioma.glioma_2_17

[14] McNee G, Eales KL, Wei W, Williams DS, Barkhuizen A, Bartlett DB, Essex S, Anandram S, Filer A, Moss PA, Pratt G, Basu S, Davies CC, Tennant DA. Citrullination of histone H3 drives IL-6 production by bone marrow mesenchymal stem cells in MGUS and multiple myeloma. Leukemia. 2017; 31:373–81. 10.1038/leu.2016.187 27400413 PMC5292682

[15] Wu DM, Wen X, Han XR, Wang S, Wang YJ, Shen M, Fan SH, Zhang ZF, Shan Q, Li MQ, Hu B, Lu J, Chen GQ, Zheng YL. Bone marrow mesenchymal stem cell-derived exosomal MicroRNA-126-3p inhibits pancreatic cancer development by targeting ADAM9. Mol Ther Nucleic Acids. 2019; 16:229–45. 10.1016/j.omtn.2019.02.022 30925451 PMC6439275

[16] Shi Y, Du L, Lin L, Wang Y. Tumour-associated mesenchymal stem/stromal cells: emerging therapeutic targets. Nat Rev Drug Discov. 2017; 16:35–52. 10.1038/nrd.2016.193 27811929

[17] Yan X, Zhang D, Wu W, Wu S, Qian J, Hao Y, Yan F, Zhu P, Wu J, Huang G, Huang Y, Luo J, Liu X, et al. Mesenchymal stem cells promote hepatocarcinogenesis via lncRNA-MUF interaction with ANXA2 and miR-34a. Cancer Res. 2017; 77:6704–16. 10.1158/0008-5472.CAN-17-1915 28947421

[18] Searles SC, Santosa EK, Bui JD. Cell-cell fusion as a mechanism of DNA exchange in cancer. Oncotarget. 2017; 9:6156–73. 10.18632/oncotarget.23715 29464062 PMC5814202

[19] Mercapide J, Rappa G, Lorico A. The intrinsic fusogenicity of glioma cells as a factor of transformation and progression in the tumor microenvironment. Int J Cancer. 2012; 131:334–43. 10.1002/ijc.26361 21858806

[20] Wang Z, Yuan Y, Zhang L, Min Z, Zhou D, Yu S, Wang P, Ju S, Jun L, Fu J. Impact of cell fusion in myeloma marrow microenvironment on tumor progression. Oncotarget. 2018; 9:30997–1006. 10.18632/oncotarget.25742 30123422 PMC6089556

[21] Melzer C, von der Ohe J, Hass R. MSC stimulate ovarian tumor growth during intercellular communication but reduce tumorigenicity after fusion with ovarian cancer cells. Cell Commun Signal. 2018; 16:67. 10.1186/s12964-018-0279-1 30316300 PMC6186086

[22] Xue J, Zhu Y, Sun Z, Ji R, Zhang X, Xu W, Yuan X, Zhang B, Yan Y, Yin L, Xu H, Zhang L, Zhu W, Qian H. Tumorigenic hybrids between mesenchymal stem cells and gastric cancer cells enhanced cancer proliferation, migration and stemness. BMC Cancer. 2015; 15:793. 10.1186/s12885-015-1780-1 26498753 PMC4620013

[23] Noubissi FK, Harkness T, Alexander CM, Ogle BM. Apoptosis-induced cancer cell fusion: a mechanism of breast cancer metastasis. FASEB J. 2015; 29:4036–45. 10.1096/fj.15-271098 26085132

[24] Huang Z, Li S, Fan W, Ma Q. Transforming growth factor β1 promotes invasion of human JEG-3 trophoblast cells via TGF-β/Smad3 signaling pathway. Oncotarget. 2017; 8:33560–70. 10.18632/oncotarget.16826 28432277 PMC5464890

[25] Zonneville J, Safina A, Truskinovsky AM, Arteaga CL, Bakin AV. TGF-β signaling promotes tumor vasculature by enhancing the pericyte-endothelium association. BMC Cancer. 2018; 18:670. 10.1186/s12885-018-4587-z 29921235 PMC6008941

[26] Wei Z, Shan Z, Shaikh ZA. Epithelial-mesenchymal transition in breast epithelial cells treated with cadmium and the role of snail. Toxicol Appl Pharmacol. 2018; 344:46–55. 10.1016/j.taap.2018.02.022 29501589 PMC5866788

[27] Guo L, Liu Z, Tang X. Overexpression of SLFN5 induced the epithelial-mesenchymal transition in human lung cancer cell line A549 through β-catenin/snail/e-cadherin pathway. Eur J Pharmacol. 2019; 862:172630. 10.1016/j.ejphar.2019.172630 31472120

[28] Seyfried TN, Huysentruyt LC. On the origin of cancer metastasis. Crit Rev Oncog. 2013; 18:43–73. 10.1615/critrevoncog.v18.i1-2.40 23237552 PMC3597235

[29] Bastida-Ruiz D, Van Hoesen K, Cohen M. The dark side of cell fusion. Int J Mol Sci. 2016; 17:638. 10.3390/ijms17050638 27136533 PMC4881464

[30] Melzer C, von der Ohe J, Hass R. Enhanced metastatic capacity of breast cancer cells after interaction and hybrid formation with mesenchymal stroma/stem cells (MSC). Cell Commun Signal. 2018; 16:2. 10.1186/s12964-018-0215-4 29329589 PMC5795285

[31] Hass R, von der Ohe J, Ungefroren H. Potential role of MSC/cancer cell fusion and EMT for breast cancer stem cell formation. Cancers (Basel). 2019; 11:1432. 10.3390/cancers11101432 31557960 PMC6826868

[32] Zhang LN, Huang YH, Zhao L. Fusion of macrophages promotes breast cancer cell proliferation, migration and invasion through activating epithelial-mesenchymal transition and Wnt/β-catenin signaling pathway. Arch Biochem Biophys. 2019; 676:108137. 10.1016/j.abb.2019.108137 31605677

[33] Mao P, Joshi K, Li J, Kim SH, Li P, Santana-Santos L, Luthra S, Chandran UR, Benos PV, Smith L, Wang M, Hu B, Cheng SY, et al. Mesenchymal glioma stem cells are maintained by activated glycolytic metabolism involving aldehyde dehydrogenase 1A3. Proc Natl Acad Sci USA. 2013; 110:8644–49. 10.1073/pnas.1221478110 23650391 PMC3666732

[34] Schwitalla S, Seide J, Keil S, Trosko J. Breast stem cells spontaneously fuse with breast cancer cells: Impacts on cancer stem cell formation? American Association for Cancer Research. 2008; 68:5007.

[35] Wang R, Chen S, Li C, Ng KT, Kong CW, Cheng J, Cheng SH, Li RA, Lo CM, Man K, Sun D. Fusion with stem cell makes the hepatocellular carcinoma cells similar to liver tumor-initiating cells. BMC Cancer. 2016; 16:56. 10.1186/s12885-016-2094-7 26846780 PMC4743091

[36] Xu MH, Gao X, Luo D, Zhou XD, Xiong W, Liu GX. EMT and acquisition of stem cell-like properties are involved in spontaneous formation of tumorigenic hybrids between lung cancer and bone marrow-derived mesenchymal stem cells. PLoS One. 2014; 9:e87893. 10.1371/journal.pone.0087893 24516569 PMC3916343

[37] Sun C, Dai X, Zhao D, Wang H, Rong X, Huang Q, Lan Q. Mesenchymal stem cells promote glioma neovascularization in vivo by fusing with cancer stem cells. BMC Cancer. 2019; 19:1240. 10.1186/s12885-019-6460-0 31864321 PMC6925905

[38] Matarredona ER, Pastor AM. Neural stem cells of the subventricular zone as the origin of human glioblastoma stem cells. Therapeutic implications. Front Oncol. 2019; 9:779. 10.3389/fonc.2019.00779 31482066 PMC6710355

[39] Fan Y, Xue W, Schachner M, Zhao W. Honokiol eliminates glioma/glioblastoma stem cell-like cells via JAK-STAT3 signaling and inhibits tumor progression by targeting epidermal growth factor receptor. Cancers (Basel). 2018; 11:22. 10.3390/cancers11010022 30587839 PMC6356849

[40] Liu HW, Su YK, Bamodu OA, Hueng DY, Lee WH, Huang CC, Deng L, Hsiao M, Chien MH, Yeh CT, Lin CM. The disruption of the β-Catenin/TCF-1/STAT3 signaling axis by 4-acetylantroquinonol B inhibits the tumorigenesis and cancer stem-cell-like properties of glioblastoma cells, in vitro and in vivo. Cancers (Basel). 2018; 10:491. 10.3390/cancers10120491 30563094 PMC6315804

[41] Yi L, Zhou X, Li T, Liu P, Hai L, Tong L, Ma H, Tao Z, Xie Y, Zhang C, Yu S, Yang X. Notch1 signaling pathway promotes invasion, self-renewal and growth of glioma initiating cells via modulating chemokine system CXCL12/CXCR4. J Exp Clin Cancer Res. 2019; 38:339. 10.1186/s13046-019-1319-4 31382985 PMC6683584

[42] Hu P, Li S, Tian N, Wu F, Hu Y, Li D, Qi Y, Wei Z, Wei Q, Li Y, Yin B, Jiang T, Yuan J, et al. Acidosis enhances the self-renewal and mitochondrial respiration of stem cell-like glioma cells through CYP24A1-mediated reduction of vitamin D. Cell Death Dis. 2019; 10:25. 10.1038/s41419-018-1242-1 30631035 PMC6328565

[43] Tao Z, Li T, Ma H, Yang Y, Zhang C, Hai L, Liu P, Yuan F, Li J, Yi L, Tong L, Wang Y, Xie Y, et al. Autophagy suppresses self-renewal ability and tumorigenicity of glioma-initiating cells and promotes Notch1 degradation. Cell Death Dis. 2018; 9:1063. 10.1038/s41419-018-0957-3 30337536 PMC6194143

[44] Bao S, Wu Q, McLendon RE, Hao Y, Shi Q, Hjelmeland AB, Dewhirst MW, Bigner DD, Rich JN. Glioma stem cells promote radioresistance by preferential activation of the DNA damage response. Nature. 2006; 444:756–60. 10.1038/nature05236 17051156

[45] Noh H, Zhao Q, Yan J, Kong LY, Gabrusiewicz K, Hong S, Xia X, Heimberger AB, Li S. Cell surface vimentin-targeted monoclonal antibody 86C increases sensitivity to temozolomide in glioma stem cells. Cancer Lett. 2018; 433:176–85. 10.1016/j.canlet.2018.07.008 29991446 PMC6086585

[46] Marín-Ramos NI, Thein TZ, Cho HY, Swenson SD, Wang W, Schönthal AH, Chen TC, Hofman FM. NEO212 inhibits migration and invasion of glioma stem cells. Mol Cancer Ther. 2018; 17:625–37. 10.1158/1535-7163.MCT-17-0591 29440289 PMC5935548

[47] Darragh LB, Oweida AJ, Karam SD. Overcoming resistance to combination radiation-immunotherapy: a focus on contributing pathways within the tumor microenvironment. Front Immunol. 2019; 9:3154. 10.3389/fimmu.2018.03154 30766539 PMC6366147

[48] Zhao S, Ren S, Jiang T, Zhu B, Li X, Zhao C, Jia Y, Shi J, Zhang L, Liu X, Qiao M, Chen X, Su C, et al. Low-dose apatinib optimizes tumor microenvironment and potentiates antitumor effect of PD-1/PD-L1 blockade in lung cancer. Cancer Immunol Res. 2019; 7:630–43. 10.1158/2326-6066.CIR-17-0640 30755403

[49] Liubomirski Y, Lerrer S, Meshel T, Rubinstein-Achiasaf L, Morein D, Wiemann S, Körner C, Ben-Baruch A. Tumor-stroma-inflammation networks promote pro-metastatic chemokines and aggressiveness characteristics in triple-negative breast cancer. Front Immunol. 2019; 10:757. 10.3389/fimmu.2019.00757 31031757 PMC6473166

[50] Yi Y, Zeng S, Wang Z, Wu M, Ma Y, Ye X, Zhang B, Liu H. Cancer-associated fibroblasts promote epithelial-mesenchymal transition and EGFR-TKI resistance of non-small cell lung cancers via HGF/IGF-1/ANXA2 signaling. Biochim Biophys Acta Mol Basis Dis. 2018; 1864:793–803. 10.1016/j.bbadis.2017.12.021 29253515

[51] Sundar IK, Li D, Rahman I. Small RNA-sequence analysis of plasma-derived extracellular vesicle miRNAs in smokers and patients with chronic obstructive pulmonary disease as circulating biomarkers. J Extracell Vesicles. 2019; 8:1684816. 10.1080/20013078.2019.1684816 31762962 PMC6848892

[52] de Almeida BC, Dos Anjos LG, Uno M, Cunha IW, Soares FA, Baiocchi G, Baracat EC, Carvalho KC. Let-7 miRNA’s expression profile and its potential prognostic role in uterine leiomyosarcoma. Cells. 2019; 8:1452. 10.3390/cells8111452 31744257 PMC6912804

[53] Jia M, Shi Y, Li Z, Lu X, Wang J. MicroRNA-146b-5p as an oncomiR promotes papillary thyroid carcinoma development by targeting CCDC6. Cancer Lett. 2019; 443:145–56. 10.1016/j.canlet.2018.11.026 30503553

[54] Li Y, Zhang H, Dong Y, Fan Y, Li Y, Zhao C, Wang C, Liu J, Li X, Dong M, Liu H, Chen J. MiR-146b-5p functions as a suppressor miRNA and prognosis predictor in non-small cell lung cancer. J Cancer. 2017; 8:1704–16. 10.7150/jca.16961 28775790 PMC5535726

[55] Zhang HM, Li Q, Zhu X, Liu W, Hu H, Liu T, Cheng F, You Y, Zhong Z, Zou P, Li Q, Chen Z, Guo AY. miR-146b-5p within BCR-ABL1-positive microvesicles promotes leukemic transformation of hematopoietic cells. Cancer Res. 2016; 76:2901–11. 10.1158/0008-5472.CAN-15-2120 27013199

[56] Al-Khalaf HH, Aboussekhra A. MicroRNA-141 and microRNA-146b-5p inhibit the prometastatic mesenchymal characteristics through the RNA-binding protein AUF1 targeting the transcription factor ZEB1 and the protein kinase AKT. J Biol Chem. 2014; 289:31433–47. 10.1074/jbc.M114.593004 25261470 PMC4223342

[57] Aihara T, Miyoshi Y, Koyama K, Suzuki M, Takahashi E, Monden M, Nakamura Y. Cloning and mapping of SMARCA5 encoding hSNF2H, a novel human homologue of drosophila ISWI. Cytogenet Cell Genet. 1998; 81:191–93. 10.1159/000015027 9730600

[58] Gigek CO, Lisboa LC, Leal MF, Silva PN, Lima EM, Khayat AS, Assumpção PP, Burbano RR, Smith Mde A. SMARCA5 methylation and expression in gastric cancer. Cancer Invest. 2011; 29:162–66. 10.3109/07357907.2010.543365 21261476

[59] Stopka T, Zakova D, Fuchs O, Kubrova O, Blafkova J, Jelinek J, Necas E, Zivny J. Chromatin remodeling gene SMARCA5 is dysregulated in primitive hematopoietic cells of acute leukemia. Leukemia. 2000; 14:1247–52. 10.1038/sj.leu.2401807 10914549

[60] Reis ST, Timoszczuk LS, Pontes-Junior J, Viana N, Silva IA, Dip N, Srougi M, Leite KR. The role of micro RNAs let7c, 100 and 218 expression and their target RAS, C-MYC, BUB1, RB, SMARCA5, LAMB3 and ki-67 in prostate cancer. Clinics (Sao Paulo). 2013; 68:652–57. 10.6061/clinics/2013(05)12 23778407 PMC3654318

[61] Leite KR, Morais DR, Reis ST, Viana N, Moura C, Florez MG, Silva IA, Dip N, Srougi M. MicroRNA 100: a context dependent miRNA in prostate cancer. Clinics (Sao Paulo). 2013; 68:797–802. 10.6061/clinics/2013(06)12 23778488 PMC3674267

[62] Jin Q, Mao X, Li B, Guan S, Yao F, Jin F. Overexpression of SMARCA5 correlates with cell proliferation and migration in breast cancer. Tumour Biol. 2015; 36:1895–902. 10.1007/s13277-014-2791-2 25377162

[63] Li Z, Zhou Y, Yang G, He S, Qiu X, Zhang L, Deng Q, Zheng F. Using circular RNA SMARCA5 as a potential novel biomarker for hepatocellular carcinoma. Clin Chim Acta. 2019; 492:37–44. 10.1016/j.cca.2019.02.001 30716279

[64] Tian JD, Liang L. Involvement of circular RNA SMARCA5/microRNA-620 axis in the regulation of cervical cancer cell proliferation, invasion and migration. Eur Rev Med Pharmacol Sci. 2018; 22:8589–98. 10.26355/eurrev_201812_16622 30575898

[65] Dong J, Dai XL, Lu ZH, Fei XF, Chen H, Zhang QB, Zhao YD, Wang ZM, Wang AD, Lan Q, Huang Q. Incubation and application of transgenic green fluorescent nude mice in visualization studies on glioma tissue remodeling. Chin Med J (Engl). 2012; 125:4349–54. 23253700

