# Supplementary material for: MiR-146b-5p suppresses the malignancy of GSC/MSC fusion cells by targeting SMARCA5
Source: Aging (Albany NY). 2020 Jul 6;12(13):13647–67. doi: 10.18632/aging.103489 (PMC7377863; doi:10.18632/aging.103489)
Supplement: Supplementary Figures [file aging-12-103489-s001..pdf]

SUPPLEMENTARY FIGURES

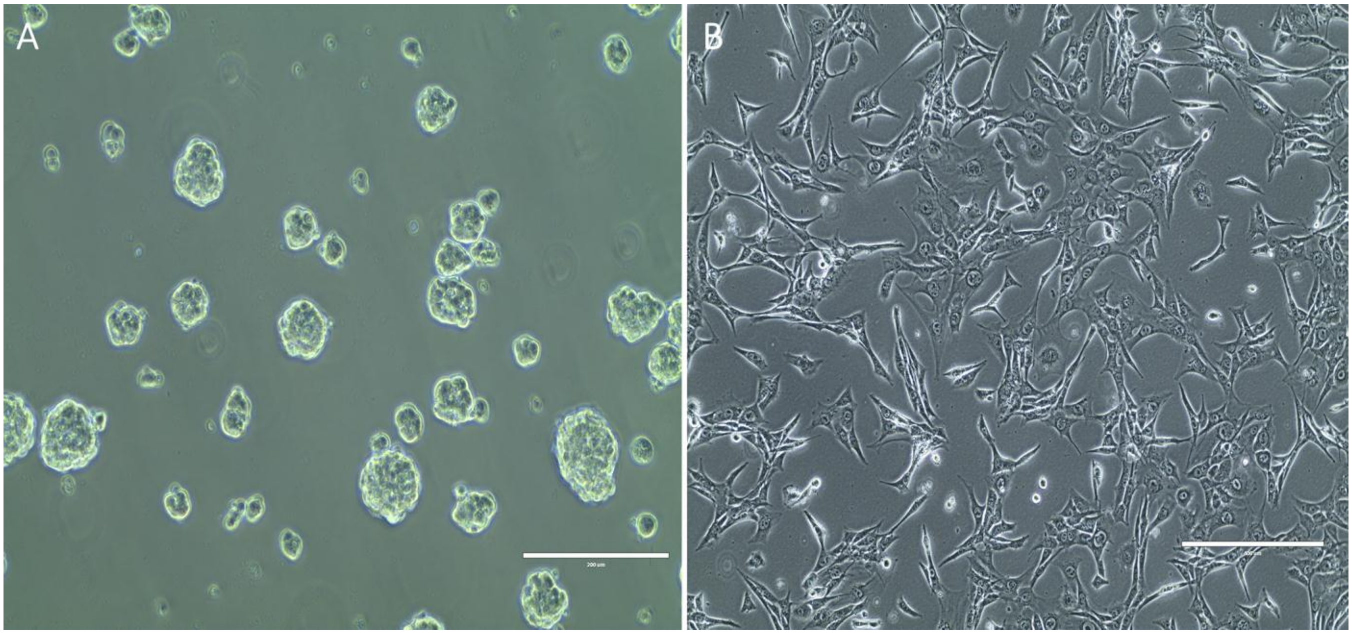

Supplementary Figure 1. The culture of GSCs-SU4 cells. Sphere-like cell clusters (**A**) and adherent growth (**B**) of GSCs-SU4 cells.

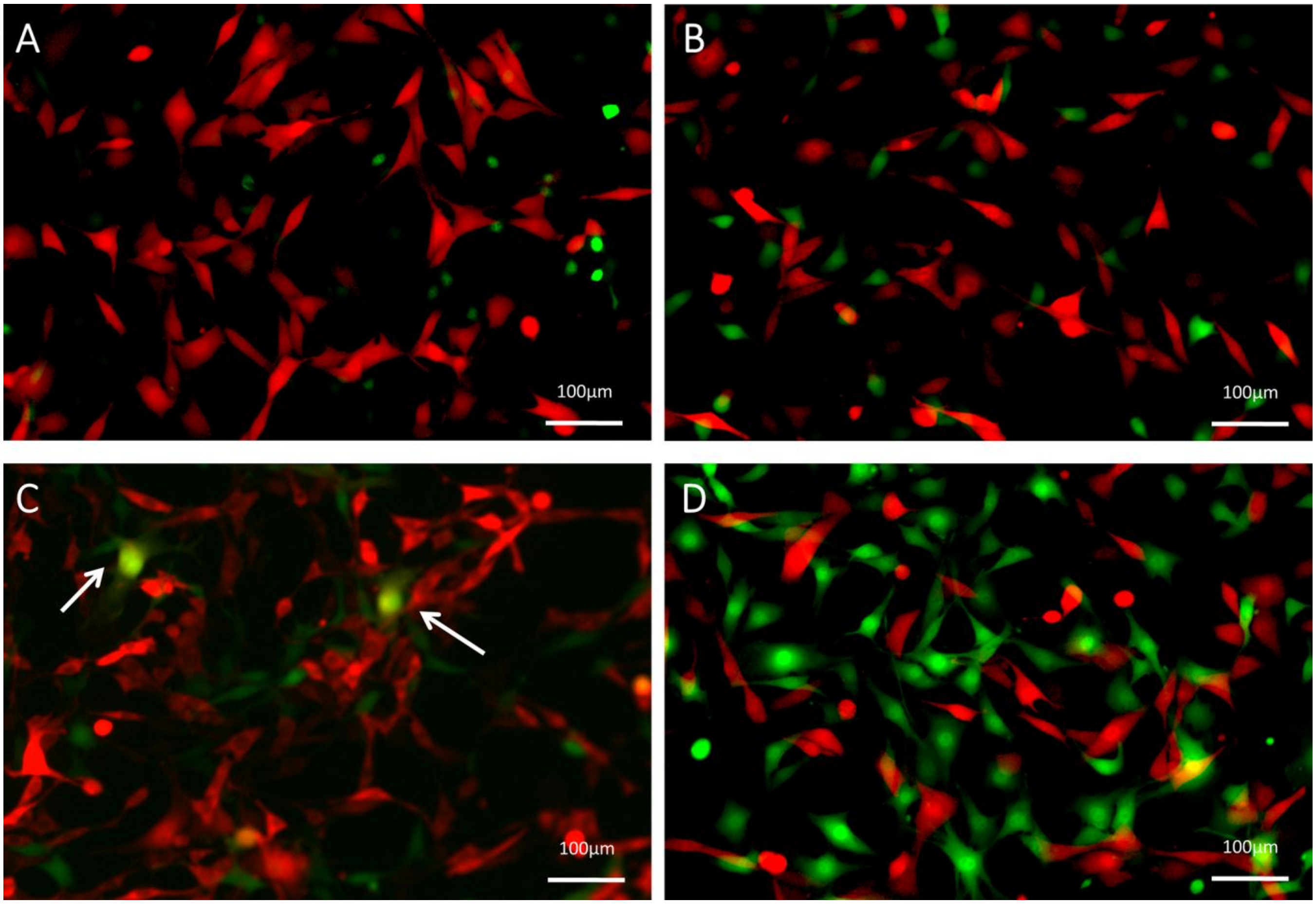

Supplementary Figure 2. Co-culture of GSCs and MSCs with different cell ratio gradients. (**A**) Co-culture of GSCs and MSCs at the ratio of 1:1. (**B**) Co-culture of GSCs and MSCs at the ratio of 1:10. (**C**) Co-culture of GSCs and MSCs at the ratio of 1:20 (RFP+/GFP+ cells (arrows)). (**D**) Co-culture of GSCs and MSCs at the ratio of 1:40.

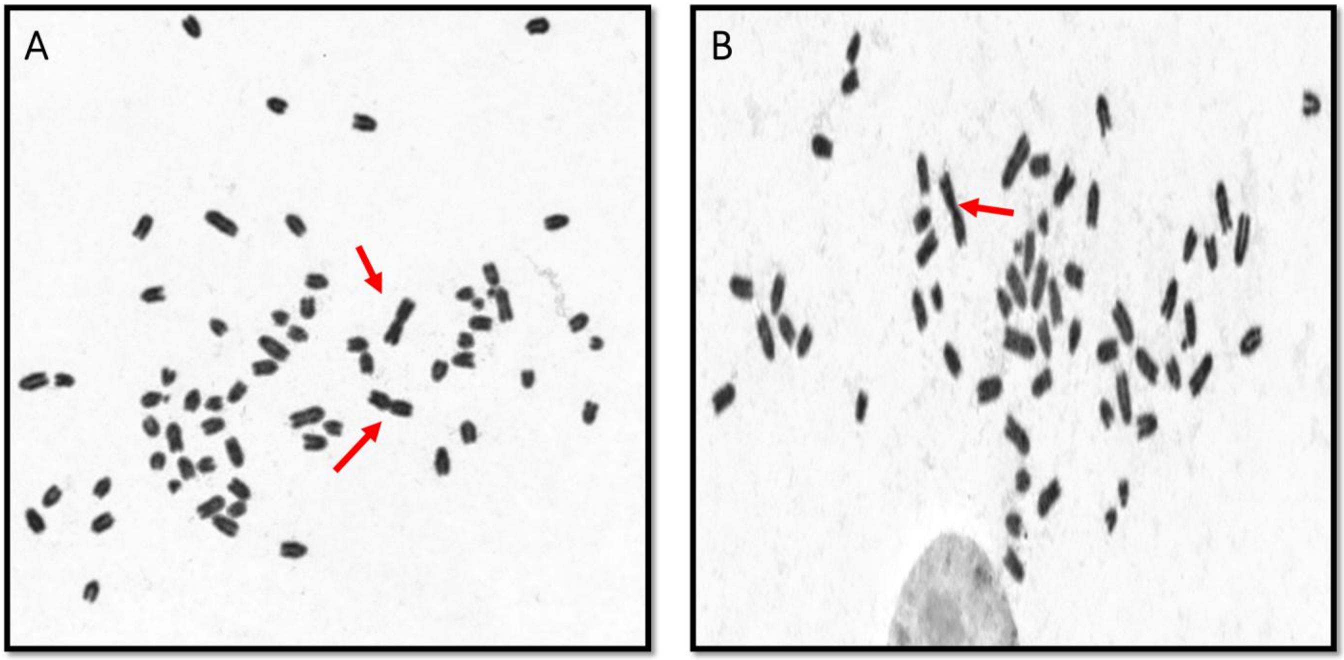

Supplementary Figure 3. The karyotype of the GSCs/MSCs fusion cells. (**A**) Fusion cells are unstable with frequent loss of human chromosomes (arrows). (**B**) Only one human chromosomes(arrows) were left with subculture of the fusion cells.

A

miRNA Name	hsa-miR-18a-3p	mmu-miR-18a-3p
miRNA Sequence	ACUGCCCUAAGUGCUCCUUCUGG	ACUGCCCUAAGUGCUCCUUCUG
miRNA Name	hsa-miR-191-3p	mmu-miR-191-3p
miRNA Sequence	GCUGCGCUUGGAUUUCGUCCCC	GCUGCACUUGGAUUUCGUUCCC
miRNA Name	hsa-miR-27b-5p	mmu-miR-27b-5p
miRNA Sequence	AGAGCUUAGCUGAUUGGUGAAC	AGAGCUUAGCUGAUUGGUGAAC
miRNA Name	hsa-miR-26b-3p	mmu-miR-26b-3p
miRNA Sequence	CCUGUUCUCCAUUACUUGGCU	CCUGUUCUCCAUUACUUGGCUC
miRNA Name	hsa-miR-532-3p	mmu-miR-532-3p
miRNA Sequence	CCUCCCACACCCAAGGCUUGCA	CCUCCCACACCCAAGGCUUGCA
miRNA Name	hsa-miR-582-3p	mmu-miR-582-3p
miRNA Sequence	UAACUGGUUGAACAACUGAACC	UAACCUGUUGAACAACUGAAC
miRNA Name	hsa-miR-148a-5p	mmu-miR-148a-5p
miRNA Sequence	AAAGUUCUGAGACACUCCGACU	AAAGUUCUGAGACACUCCGACU
miRNA Name	hsa-miR-29c-3p	mmu-miR-29c-3p
miRNA Sequence	UAGCACCAUUUGAAAUCGGUUA	UAGCACCAUUUGAAAUCGGUUA
miRNA Name	hsa-miR-15b-5p	mmu-miR-15b-5p
miRNA Sequence	UAGCAGCACAUCAUGGUUUACA	UAGCAGCACAUCAUGGUUUACA

B

miRNA Name	hsa-miR-23b-3p	mmu-miR-23b-3p
miRNA Sequence	AUCACAUUGCCAGGGAUUACCAC	AUCACAUUGCCAGGGAUUACC
miRNA Name	hsa-miR-532-5p	mmu-miR-532-5p
miRNA Sequence	CAUGCCUUGAGUGUAGGACCGU	CAUGCCUUGAGUGUAGGACCGU
miRNA Name	hsa-miR-146a-5p	mmu-miR-146a-5p
miRNA Sequence	UGAGAACUGAAUUCCAUGGGUU	UGAGAACUGAAUUCCAUGGGUU
miRNA Name	hsa-miR-27b-3p	mmu-miR-27b-3p
miRNA Sequence	UUCACAGUGGCUAAGUUCUGC	UUCACAGUGGCUAAGUUCUGC
miRNA Name	hsa-miR-92a-3p	mmu-miR-92a-3p
miRNA Sequence	UAUUGCACUUGUCCCGGCCUGU	UAUUGCACUUGUCCCGGCCUG
miRNA Name	hsa-miR-142-5p	mmu-miR-142-5p
miRNA Sequence	CAUAAAGUAGAAAGCACUACU	CAUAAAGUAGAAAGCACUACU
miRNA Name	hsa-miR-26a-5p	mmu-miR-26a-5p
miRNA Sequence	UUCAAGUAAUCCAGGAUAGGCU	UUCAAGUAAUCCAGGAUAGGCU
miRNA Name	hsa-miR-146b-5p	mmu-miR-146b-5p
miRNA Sequence	UGAGAACUGAAUUCCAUAGGCUG	UGAGAACUGAAUUCCAUAGGCU
miRNA Name	hsa-miR-181a-5p	mmu-miR-181a-5p
miRNA Sequence	AACAUUCAACGCUGUCGGUGAGU	AACAUUCAACGCUGUCGGUGAGU

Supplementary Figure 4. The sequence of these miRNAs is the same between human and mouse. The most significant differentially expressed miRNAs that sharing between human and mouse can be selected as the candidate miRNAs.

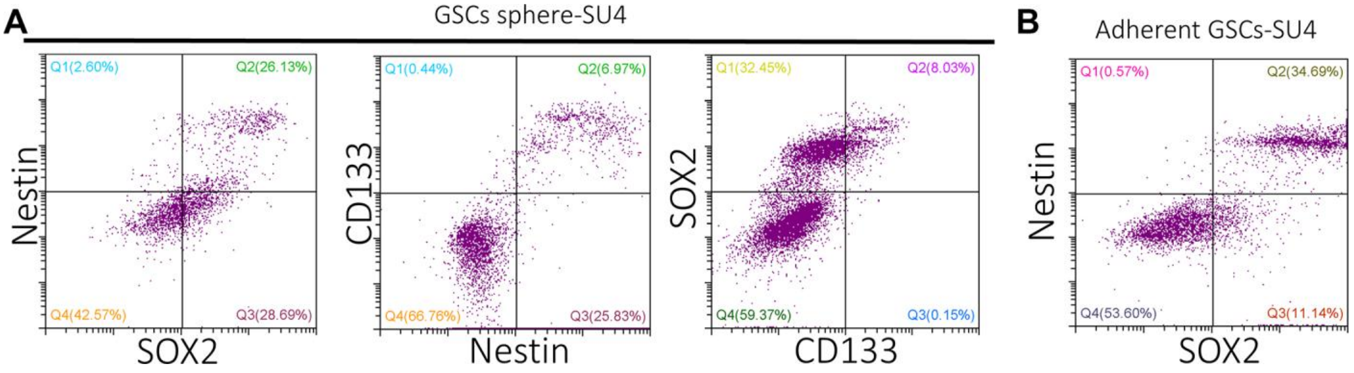

Supplementary Figure 5. Co-expression of GSCs markers in GSC-SU4 cells by flow cytometry. There was no significant difference in the proportion of adherent cultured GSCs with positive markers (**B**), compared with sphere culture (**A**).
